# Complete Genome Sequence of Rhodococcus erythropolis JCM 2895, an Antibiotic Protein-Producing Strain

**DOI:** 10.1128/mra.00682-22

**Published:** 2022-11-02

**Authors:** Wataru Kitagawa, Miyako Hata

**Affiliations:** a Bioproduction Research Institute, National Institute of Advanced Industrial and Technology, Sapporo, Hokkaido, Japan; b Graduate School of Agriculture, Hokkaido University, Sapporo, Hokkaido, Japan; University of Maryland School of Medicine

## Abstract

The complete genome sequence of Rhodococcus erythropolis JCM 2895, an antibiotic protein-producing strain, was determined. It consists of a 6,455,263-bp chromosome, one linear plasmid (pR09L01 [227,989 bp]), and three circular plasmids (pR09C01 [79,600 bp], pREC01 [5,420 bp], and pREC02 [5,444 bp]).

## ANNOUNCEMENT

The genus *Rhodococcus* is a member of the phylum *Actinobacteria* and has been well studied for its great ability to degrade persistent chemicals such as polychlorinated biphenyls and dioxins (1–4). Recently, some antibiotic-producing *Rhodococcus* strains have also been reported (5–7). Rhodococcus erythropolis strain JCM 2895, which was originally isolated as a hydrocarbon utilizer (8), has been reported to produce extracellular bacteriocin-like protein RapA (9). In this study, genome sequence analysis of this strain was performed.

Strain JCM 6824 was cultivated in liquid LB medium for 20 h at 28°C, and its genomic DNA was extracted using a conventional phenol-chloroform method. The nucleotide sequence of the genomic DNA was determined by using three sequencing technologies, namely, Illumina HiSeq, Roche 454 GS FLX+, and Pacific Biosciences (PacBio) RS II; a single genomic preparation was used for all three. When preparing the sequencing libraries and when filtering and assembling the reads, developers’ default protocols and parameters were used for all kits and software. Illumina HiSeq sequencing, using the TruSeq DNA library preparation kit v2, yielded a total of 3,453 Mb distributed in 34,190,088 reads, which generated 114 contigs (*N*_50_, 397,738 bp) with CASAVA v1.8.1 base-calling/filtering software (Illumina) and Velvet v1.2.01 assembly software. Roche 454 GS FLX+ sequencing, using the GS FLX Titanium rapid library preparation kit, yielded a total of 88 Mb distributed in 139,426 reads (average read length, 631 bases), which generated 161 contigs (*N*_50_, 119,954 bp) with GS Run Processor v2.8 base-calling/filtering software (Roche) and GS De Novo Assembler v2.8 software. For PacBio sequencing, DNA was sheared using a g-TUBE (Covaris) to around 20 kb, and size selection was performed with BluePippin (Sage Science) with a cutoff value of 10 kb. PacBio sequencing, using the SMRTbell template preparation kit v1.0, yielded a total of 1,184 Mb distributed in 180,093 reads (average read length, 6,579 bases), which generated 8 contigs (*N*_50_, 6,471,546 bp) with single-molecule real-time (SMRT) Analysis v2.2 integrated filtering and assembling software (PacBio). The contigs from these three methods were compared and combined with GenomeMatcher software v3.0.4 (10), and the complete nucleotide sequences of four circular replicons and one incomplete linear replicon were determined. Circularity was confirmed by manual PCR followed by sequencing of the junction, and the termini of the linear replicon were manually analyzed with a terminal-fragment cloning method (11).

The R. erythropolis JCM 2895 genome consists of one circular chromosome (6,455,263 bp), one linear plasmid (pR09L01 [227,989 bp]), and three circular plasmids (pR09C01 [79,600 bp], pREC01 [5,420 bp], and pREC02 [5,444 bp]). The G+C contents are 59 to 62%. Genes were annotated using the DDBJ Fast Annotation and Submission Tool (DFAST) (12), with the rotate option so that the *dnaA* gene comes first. The genome contains 6,318 putative coding sequences, 60 tRNAs, and 5 *rrn* operons. The genome information will facilitate understanding of the ecological features of this strain.

### Data availability.

This whole-genome sequence has been deposited in DDBJ under the accession no. AP018733 to AP018736 and AB893594. The raw sequence reads have been deposited in the DRA under the accession no. DRA014265.
